# Incidence of *Gardnerella vaginalis, Candida sp* and human papilloma virus in cytological smears

**DOI:** 10.1590/S1516-31802000000400006

**Published:** 2000-07-07

**Authors:** Eddie Fernando Candido Murta, Maria Azniv Hazarabedian de Souza, Eduardo Araújo, Sheila Jorge Adad

**Keywords:** Human papilloma virus, *Gardnerella vaginalis*, *Candida sp*, Cytological smear, Papilomavírus humano, Gardnerella vaginalis, Candida sp, Exame citológico

## Abstract

**CONTEXT:**

In spite of the wide-ranging literature on the microbiology of normal and abnormal flora of the vagina, there are few studies on the relationship between human papilloma virus (HPV) and other vaginal microorganisms.

**OBJECTIVE:**

To analyze the frequency of infection by human papilloma virus (HPV) and other agents like *Candida sp., Gardnerella vaginalis* and *Trichomonas vaginalis* in cytological smears.

**DESIGN STUDY:**

Retrospective study

**SETTING:**

A public tertiary referral center.

**SAMPLE:**

An analysis of 17,391 cytologies from outpatients seen between January 1997 and August 1998. The control group was made up of patients in the same age group and same period with no cytological evidence of HPV infection. Patients with a diagnosis of cervical intraepithelial neoplasia (CIN) II or III were excluded from this analysis.

**MAIN MEASUREMENTS:**

The diagnosis of HPV infection was made in accordance with the criteria of Schneider et al. and the diagnosis of *Gardnerella vaginalis* was made with a finding of clue cells.

**RESULTS:**

390 (2.24%) had alterations consistent with infection by HPV, sometimes associated with CIN I. The results showed that *Gardnerella vaginalis* was the most frequent agent in women with HPV infection (23.6% versus 17.4%; P <0.05), while in the control group the most frequent agent was *Candida sp*. (23.9% versus 13.8%; p <0.001).

**CONCLUSION:**

In spite of this study being based solely on cytological criteria, in which specific HPV and Gardnerella diagnostic tests were not used, the cytological smear is widely used in clinical practice and the data presented in this investigation show that there is an association between *Gardnerella vaginalis* and HPV infection. It remains to be established whether the microorganisms favor each other.

## RESUMO

**CONTEXTO:**

Apesar da extensa literatura sobre a microbiologia da flora vaginal normal e anormal, há poucos trabalhos sobre a relação entre papilomavírus humano (HPV) e outros microorganismos vaginais.

**OBJETIVO:**

Analisar a freqüência de infecção por HPV e outros agentes como *Candida sp., Gardnerella vaginalis* e *Trichomonas vaginalis* no exame citológico.

**TIPO DE ESTUDO:**

Estudo retrospectivo.

**LOCAL:**

Centro público de referência terciária.

**AMOSTRA:**

17.391 citologias de pacientes ambulatoriais atendidas entre janeiro de 1997 e agosto de 1998. O grupo controle foi composto por mulheres da mesma idade sem sinais citológicos de infecção por HPV. As pacientes com diagnóstico de neoplasia intraepitelial cervical (NIC) II ou III foram excluídas desta análise.

**VARIÁVEIS ESTUDADAS:**

O diagnóstico citológico de infecção por HPV foi feito segundo os critérios de Schneider, et al. e o diagnóstico de Gardnerella vaginalis foi feito pelo achado de "clue cells".

**RESULTADOS:**

390 (2,24%) tinham alterações compatíveis com infecção por HPV associada ou não a NIC I. Os resultados mostraram que a Gardnerella vaginalis foi o agente mais freqüente nas mulheres com infecção por HPV (23,6% versus 17,4%; P <0,05), enquanto no grupo controle o agente mais frequente foi a Candida sp. (23,9% versus 13,8%; P <0,001).

**CONCLUSÃO:**

Apesardeste estudo ser baseado somente em critérios citológicos, em que testes diagnósticos específicos não foram usados, o exame citológico é largamente usado na prática clínica e os dados apresentados nesta investigação mostram a associação entre a Gardnerella vaginalis e a infecção por HPV. Se os microorganismos favorecem um ao outro, isto ainda está por ser estabelecido.

## INTRODUCTION

An extensive and diverse spectrum of pathogenic and non-pathogenic organisms may be observed in vaginal microflora. In spite of the wide-ranging literature on the microbiology of normal and abnormal flora of the vagina, many questions have still not been completely answered.^1,2^ Various infectious processes in the vagina are the result of disequilibrium of this flora^1^, such as that occurring during pregnancy.^2^ The most common infections are caused by *Candida sp., Gardnerella vaginalis, Trichomonas vaginalis* and *Chlamydia trachomatis*, whose frequencies vary among non-pregnant women from 7 to 20.5%, 2 to 58%, 9 to 12% and 8 to 40%, respectively.^3-5^ Human papilloma virus (HPV) infection is also common and presents a frequency that varies from 13 to 46%, depending on the population studied and the study method used.^6,7^

Some of the epidemiological factors related to greater incidence of HPV infection are well-known: young women, especially those who start to be sexually active at a very early age, smokers, pregnant women or those using oral contraceptives.^8,9^ However, there are few studies on the relationship between HPV and other vaginal micro-organisms. The objective of this work was to study a group of women with a cytological diagnosis of HPV, to verify the frequency of others agents, especially *Candida sp., Gardnerella vaginalis* and *Trichomonas vaginalis*.

## METHODS

An analysis was made of 17,391 cytologies from outpatients attended to in the period from January 1997 until August 1998. Of the total of 17,391 cytologies, 390 (2.24%) had a cytological report diagnosing HPV infection, sometimes associated with cervical intraepithelial neoplasia (CIN) grade I (low-grade squamous lesion). Patients with a diagnosis of CIN II or CIN III were excluded from this analysis.

The diagnosis of HPV infection was made in accordance with the criteria of Schneider et al.^10^ which are based on the presence of classic coilocytosis or the presence of six or more of the nine non-classic criteria (mild coilocytosis, mild dekeratosis, cytoplasmic clarification, keratohyalin granules, cytoplasmic striation, dekeratotic fusiform cells, nuclear hyperchromatism, bi- or multi-nucleation and perinuclear halo). The diagnosis of *Gardnerella vaginalis* was made with a finding of clue cells. The control group was made up of patients in the same age group and same period with no cytological evidence of HPV infection. The concomitant presence of fungus (*Candida sp*.), bacteria (*Gardnerella* and *Actinomyces*), protozoans (*Trichomonas vaginalis*) and infection by Herpes virus was analyzed for both groups.

The X^2^ test was used for statistical analysis with the significance level set at less than 0.05.

## RESULTS

A distribution of patients with HPV infection is shown in Table 1, matched with the control group according to age group. It can be seen that 83.6% of the patients with HPV infection were aged 40 or under, and 38.7% were between 21 and 30. In the group with HPV infection, other agents were detected in 146 (37.4%) of the 390 cases and in the control group, in 167 (42.2%) of the 396 cases. However, this difference was not considered statistically significant (χ^2^ = 1.839; Yates correction = 1.647). There were 61 (15.6%) pregnant patients in the group with HPV and 70 (17.6%) in the control group. Table 2 shows the frequency of the most common infectious agents in cytological exams in the group with HPV, in relation to the control. It may be observed that *Gardnerella vaginalis* was the most frequent agent in the group with HPV infection, while in the control group the most common agent was *Candida sp*. There was no statistically significant difference between the groups in relation to infection by *Trichomonas vaginalis*. No cases of Herpes virus and/or Actinomyces were observed in any of the patients in the study groups.

**Table 1:** Distribution of patients with cytological signs of HPV, compared to the control group, according to age group

Age group (years)	HPV		Control	
	n	%	n	%
≤ 20	99	25.4	100	25.3
21-30	151	38.7	153	38.6
31-40	76	19.5	77	19.5
41-50	34	8.7	35	8.8
51-60	18	4.6	18	4.5
≥ 61	12	3.1	13	3.3
**TOTAL**	**390**	**100**	**396**	**100**

**Table 2:** Frequency of infection by various agents in the group of patients with cytological signs of HPV infection, in relation to the control group

Cytological findings	HPV		Control		^p^
	n	%	n	%	
*Candida*	*47*	*12.0*	*88*	*22.2*	*<0.001*
*Gardnerella*	*83*	*21.3*	*59*	*14.9*	*<0.05*
*Trichomonas*	*7*	*1.8*	*9*	*2.3*	*ns*
*Candida + Gardnerella*	*7*	*1.8*	*6*	*1.5*	*ns*
*Candida + Trichomonas*	*0*	*0*	*1*	*0.2*	*ns*
*Gardnerella + Trichomonas*	*2*	*0.5*	*4*	*1.0*	*ns*
*Without agents*	244	62.6	229	57.8	ns
**TOTAL**	**390**	**100**	**396**	**100**	

χ^2^ test; ns = not significant.

## DISCUSSION

The presence of genital co-infections, whether sexually transmitted or not, may have importance in the cell proliferation associated with HPV. This probably occurs through an increase in moistness in the vaginal environment.^11^ Kinghorn^12^ diagnosed other genital infections in 32% of men and 61% of women who presented condylomatous lesions, with 29% of the men 28% of the women having sexually transmitted diseases (STD). In a study of 179 men and 168 women with condylomatous lesions, Carne^13^ found co-infections in 34% and 54% respectively, with 80% of the men and 51% of the women having STD. Strand et al.^14^ detected HPV infection via PCR in 30.5% of the patients (66 men and 65 women) who went to an STD clinic for consultation because of symptoms, or for treatment of some sexually transmitted disease. Voog, et al.^15^ studied the presence of *Candida* via culture in patients positive to DNA HPV, in relation to those negative to DNA HPV. A frequency of 26% in the first group and 16% in the second was found. The prevalence of *Chlamydia* was 6% in patients positive to DNA HPV, compared to 12% in patients negative to DNA HPV. Other studies have demonstrated a prevalence of *Chlamydia* varying from 12 to 18%.^16,17^ In our work, we did not analyze the frequency of *Chlamydia*, as cytology is not the method recommended for its diagnosis due to the method's low sensitivity,^18^ which would thus underestimate the number found.

Infection by *Candida* has been found in approximately 25% of patients with HPV infection.^12,15^ Voog, et al.^15^ raised the possibility that infection by *Candida* could activate latent HPV infection. In our material, cytological diagnosis of *Candida* was made in 13.8% of the cases with HPV infection and in 23.9% of the control group (P < 0.001), the inverse of the results obtained by Voog, et al.^15^ However, these authors did not age-match the groups which were positive and negative to DNA HPV by age.

Studies of vaginal flora in healthy women have demonstrated percentages of 25% for *Gardnerella vaginalis*,^4^ and 10 to 55% for *Candida sp*.^19^ In our material, independent of the set of symptoms, respectively 23.9 and 17.4% of women in the control group presented *Candida* and *Gardnerella vaginalis* upon cytological examination, while in the group with HPV the proportions were 13.8 and 23.6%. This led us to question whether women who present *Gardnerella* in the vaginal flora could have a greater predisposition to HPV infection. The risk factors for HPV infection are already well known and it is not within our knowledge that alterations in the vaginal flora could have a role in predisposition to this infection. On the other hand, there is no data in the literature indicating that HPV infection could favor the growth of *Gardnerella* in the vaginal flora. Platz-Christensen, et al.^20^ suggested that bacterial vaginosis is in some way associated with the development of CIN, i.e., as a cofactor to HPV.

In summary, in spite of this study being based solely on cytologic criteria, in which specific HPV and *Gardnerella* diagnostics tests were not used, the cytological smear is widely used in clinical practice and the greater frequency of *Gardnerella* in patients with HPV infection found in this work deserves deeper investigation. The data presented in this investigation show that there is an association between *Gardnerella vaginalis* and HPV infection. It remains to be established whether the microorganisms favor each other. Better understanding of vaginal physiology and the possible direct or indirect relation between HPV and *Gardnerella vaginalis* could lead to great strides forward in the understanding of the physiopathology of HPV infection.
